# Using Data From a Short Video Social Media Platform to Identify Emergent Monkeypox Conspiracy Theories

**DOI:** 10.1001/jamanetworkopen.2022.36993

**Published:** 2022-10-18

**Authors:** Marco Zenone, Timothy Caulfield

**Affiliations:** 1 Health Law Institute, Faculty of Law, University of Alberta, Edmonton, Alberta, Canada

## Abstract

This qualitative study investigates emergent conspiracy theories about monkeypox collected from content and metadata posted by users of a social media video app.

## Introduction

Since May 6, 2022, a monkeypox outbreak has spread to or been detected in more than 100 countries. Shortly after media coverage of the outbreak, misinformation about monkeypox was reported.^1^ The World Health Organization advised “detecting public sentiment…[to] address possible rumours and misinformation.”^2^ This qualitative study used real-time data from TikTok, a social media app allowing users to create and share short videos, to proactively identify monkeypox conspiracy theories for public health to debunk before their potential viral spread.

## Methods

To identify emergent conspiracy theories about monkeypox, content and metadata under *#monkeypox* (864 videos) on the app’s desktop version were collected using the DataMiner website scraper tool and manual retrieval of videos on May 21, 2022. We chose the app owing to its high monthly use (>1 billion users). We reviewed each English-language video for the presence of a conspiracy theory or theme related to the emerging monkeypox outbreak. We classified theories into a typology and reported their viewership and engagement metrics. M.Z. coded the videos; T.C. audited coding decisions. Ethical approval and informed consent were not needed because videos were publicly available and posted without expectation of privacy. Data were analyzed using Microsoft Excel, version 2208.

## Results

We identified 153 videos with a monkeypox conspiracy theory or theme. The estimated mean time since posting was 30.2 hours. In total, videos received 1 485 911 views (median, 2872; IQR, 1691-6697), 74 328 likes (median, 192; IQR, 93-386), 7890 comments (median, 26; IQR, 12-53), and 13 783 shares (median, 21; IQR, 8-58).

Our analysis sorted conspiracy theories into 11 types (Table). The most prominent were assertions that monkeypox was a planned pandemic introduced for power, control, or money or to instill fear (71 videos [46.4%]); content alleged that monkeypox was a purposeful repeating of COVID-19, intentionally released, or associated with COVID-19 and conspiracy theories such as “the great reset” and “one world order.”

**Table.** Monkeypox Conspiracy Themes by Characterization and Examples

Conspiracy theme	Videos, No. (%) (N = 153)	Characterization	Transcribed examples
Monkeypox is the next orchestrated pandemic	71 (46.4)	Introducing another pandemic for power, control, or money; monkeypox is fake or exaggerated; certain groups were aware of an upcoming outbreak; monkeypox was intentionally released; ties to existing conspiracy theories: “one world order” and “the great reset”	“When they couldn’t fool us with the first pandemic, so they start with the monkeypox.” “The COVID pandemic was just a test run. They’re not done. They saw how compliant we were for a flulike virus and now they’re going to release another one. And guaranteed it will be worse.” “It’s almost like they knew it was coming, like it was a giant plan, from 1 pandemic to the next, that’s all it’s going to be now guys that way they can keep all the control they want and keep everyone scared….This is a giant plan.”
Monkeypox was introduced to administer vaccines	51 (33.3)	Vaccine manufacturers knew about or were tied to monkeypox outbreak; governments’ orders and approvals for smallpox vaccines taken as evidence they knew about an upcoming outbreak; vaccines to kill or injure; distortion of why smallpox vaccines exist after disease eradication	“In November of 2021 Justin Trudeau spent millions of dollars on smallpox vaccines. It’s almost like they knew it was coming like it was a giant plan.” “It is [vaccine] made by Bavarian Nordic. In a bizarre coincidence Bavarian Nordic had a meeting this week that was planned 6 months ago about stockpiling it.” “They figured they couldn’t stick you with your poison the first time so they’re going to actually create an outbreak. But don’t worry they have everything in place to make sure you take your vaccines here really soon. Wake up.”
Bill Gates is involved in the monkeypox outbreak	28 (18.3)	Speaking about the need to prepare for the next pandemic taken as evidence of outbreak involvement; Bill Gates’ predicting the next pandemic will be smallpox or bioterror taken as evidence of his outbreak involvement; Bill Gates having financial interest in smallpox vaccines to create an outbreak	“Anybody else think it’s oddly suspicious a relatively short time after Bill Gates says there is going to be a smallpox outbreak, there is a monkeypox outbreak?” “The US has ordered 19 million vaccines for the monkeypox. They are made by Bavarian Nordic and their stocks are soaring through the roof now. The Bill and Melinda Gates Foundation had given them a few million dollars a year ago for making these vaccines but I’m sure it’s all a coincidence.” “Remembering what Bill Gates said about the next pandemic with a smirk on his face.”
Monkeypox is an excuse to give the WHO power	27 (17.6)	The WHO is looking to take over the sovereignty of countries; the WHO will be able to override country-specific laws and introduce lockdowns and related measures; the timing of monkeypox with the pandemic treaty was intentional	“Do people not find it ironic that on May 22 WHO was looking to take over and yesterday this came out, outbreak of monkeypox growing in Europe and now detected in the US…interesting timing if I do say so myself.” “[I]t just so happens right before the WHO wants to meet for this an outbreak of monkeypox is coming out. Coincidence I think not. They’re trying to force the new world order…”
A car crash with monkeys escaping led to the monkeypox outbreak	22 (14.4)	News coverage of an escaped monkey tied to release of smallpox and/or monkeypox; allegations that public health agencies covered up escape of monkeys; monkeys were released by pandemic planners	“Why is nobody correlating the monkey crash to the monkeypox?” “Remember when the 100 monkeys got loose and couldn’t find that one? It’s almost like they knew it was coming.” “Were they being careless on purpose, are they going to use this as a perfect cover-up as to why certain people are getting monkeypox? I don’t know. Should we trust the CDC? Ugh, you do what you want with this information.”
Monkeypox is a bioterror attack	22 (14.4)	Monkeypox was made in a laboratory; monkeypox was released intentionally; Russian involvement in a bioterror attack	“Stop taking sh-t out of your lab and releasing it. Maybe think of that how gullible are you…sheeple.” “Monkeypox virus is a Russian chemical weapon.”
Germ games (simulated pandemic preparation exercises) are proof of a planned pandemic	16 (10.5)	Germ game dates match with monkeypox outbreak; germ game exercises included monkeypox	“This was part of a report published by the NTI on November 23, 2021. They always tell us what they’re going to do next. Download the full report while you can.”
Monkeypox is a sign of the end of the world	7 (4.6)	Religious rapture; end of the world	“[F]irst it was COVID and now it’s monkeypox….We are in the birth pains and if you don’t know Jesus it’s best you get to know him now because he’s coming soon.”
Lost bottles of smallpox led to the monkeypox outbreak	6 (3.9)	Lost or misplaced vials containing smallpox were linked to the release of smallpox and/or monkeypox	“They like to expose their plans. Did everybody forget they found those weird smallpox vials that were spotted at the Maverick facility near Philadelphia…I mean it would just be a coincidence, right? It’s not like the FBI and the CDC were investigating the questionable vials that were found. And it wasn’t like there was 15 vials and some were labeled smallpox?”
COVID-19 vaccine damage hurt immunity or had adverse effects leading to the monkeypox outbreak	5 (3.3)	COVID-19 vaccines led to monkeypox; COVID-19 vaccines were responsible for lowered immunity to monkeypox	“Monkeypox outbreak in Europe. Monkeypox is actually a rare infection and not particularly infectious. But some severe cases have been reported. It’s almost like immune systems aren’t working so well. What do you think is going on there?” “How many of the people that contracted monkeypox are also vaccinated. We are approaching a time now where enough people have gotten the vaccine and enough time has passed so now we are starting to see some of the long-term effects.”
Monkeypox was introduced for political purposes	5 (3.3)	Monkeypox was released or exaggerated purposefully by politicians for political support	“I can’t be the only one that notices there’s going to be some type of sickness outbreak before each election cycle these days. Monkeypox c’mon man. Here we go again.”

Abbreviations: CDC, Centers for Disease Control and Prevention; FBI, Federal Bureau of Investigation; NTI, Nuclear Threat Initiative; WHO, World Health Organization.

Fifty-one videos (33.3%) were related to vaccines and asserted monkeypox was an excuse to administer or mandate vaccines worldwide. Vaccine manufacturers and governments were accused of knowing of an upcoming outbreak or having a role in creating the outbreak. Content alleged that government approval or orders of smallpox vaccines were proof of involvement. Five videos (3.3%) alleged that COVID-19 vaccines were the reason for or contributed to the outbreak. The World Health Organization was accused of involvement in the outbreak as an excuse to pass the May 2022 global pandemic treaty (27 [17.6%]) and erode the sovereignty of countries, override national laws, and seek broader power.

The commentary of Bill Gates predicting or addressing the need to prepare for the next pandemic was taken as evidence of his involvement (28 videos [18.3%]). Specifically, Gates’ comments about a possible bioterror attack or laboratory-created pandemic were cited, with some allegations of Russian government involvement (22 [14.4%]). Simulated pandemic preparation exercises were also taken as evidence of a preplanned pandemic (16 [10.5%]).

Several conspiracy theories tied unrelated news events to the cause and/or cover-up of the monkeypox outbreak. An escape of monkeys in the US, as covered in a news story, was cited as potentially leading to the monkeypox outbreak (22 videos [14.4%]). Lost and found vials of smallpox had similar allegations (6 [3.9%]). Other videos suggested monkeypox was a symptom of an upcoming religious rapture (7 [4.6%]) or was introduced for political purposes (5 [3.3%]).

## Discussion

Our results demonstrated the potential use of real-time social media data to identify and understand conspiracy theories before their viral spread. This is particularly important during the information-gathering phase of infectious outbreaks.^3^ The COVID-19 pandemic has shown the challenges of viral misinformation^4,5^ and the need to proactively deter it.^6^ A limitation was that we included only videos in English under 1 hashtag; there are likely videos with conspiracy theories in other languages or different hashtags. Public health experts may consider greater attention to and investment in monitoring the online environment.
